# Supplementary figures and images for: Short-wavelength-sensitive 2 (Sws2) visual photopigment models combined with atomistic molecular simulations to predict spectral peaks of absorbance
Source: PLoS Comput Biol. 2020 Oct 21;16(10):e1008212. doi: 10.1371/journal.pcbi.1008212 (PMC7605715; doi:10.1371/journal.pcbi.1008212)

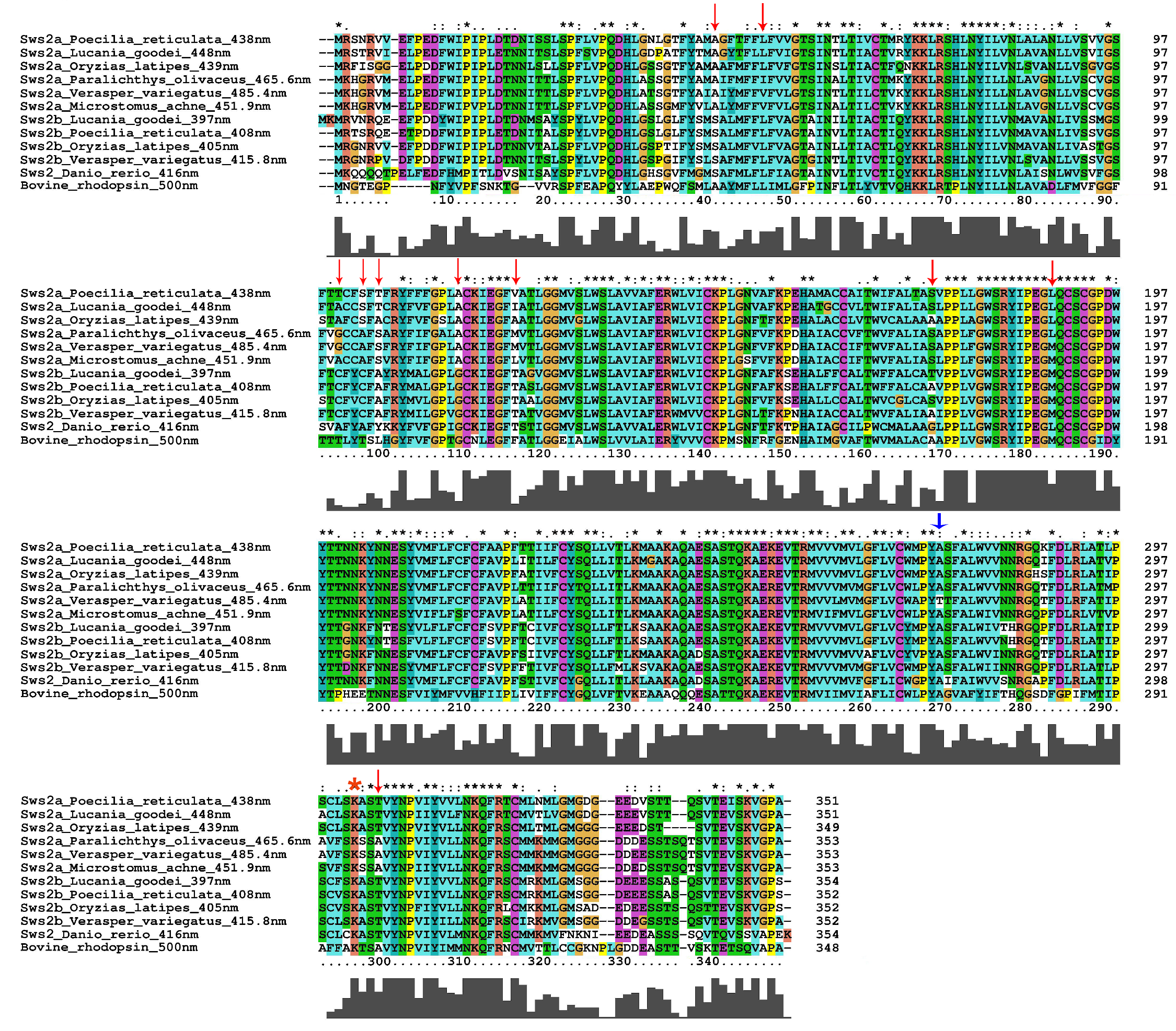

Supplement: S1 Fig — Red arrows indicate amino acid positions (using numbering standardized to bovine rod opsin) highlighted by Cortesi et al. (2015) [22] that were identified as likely to have contributed to the functional diversification of teleost Sws2 opsin sequences, particularly those that distinguish Sws2b photopigments (violet-sensitive) from the more long-wavelength-shifted Sws2a photopigments (blue-sensitive). The blue arrow indicates position 269, that is likely to be the spectral tuning site that mediates a shift in the λmax value of V. variegatus Sws2a to 485 nm [23]. A large orange asterisk (*) depicts a conserved lysine (K) residue at position 296 that is required for the formation of the Schiff-base linkage to the retinal chromophore. The gray bars below each alignment indicate a quality score, such that lower scores correspond to greater amino acid variability in the column. All species and photopigment subclasses are followed by experimentally measured λmax values. (TIF) [file pcbi.1008212.s001.tif]

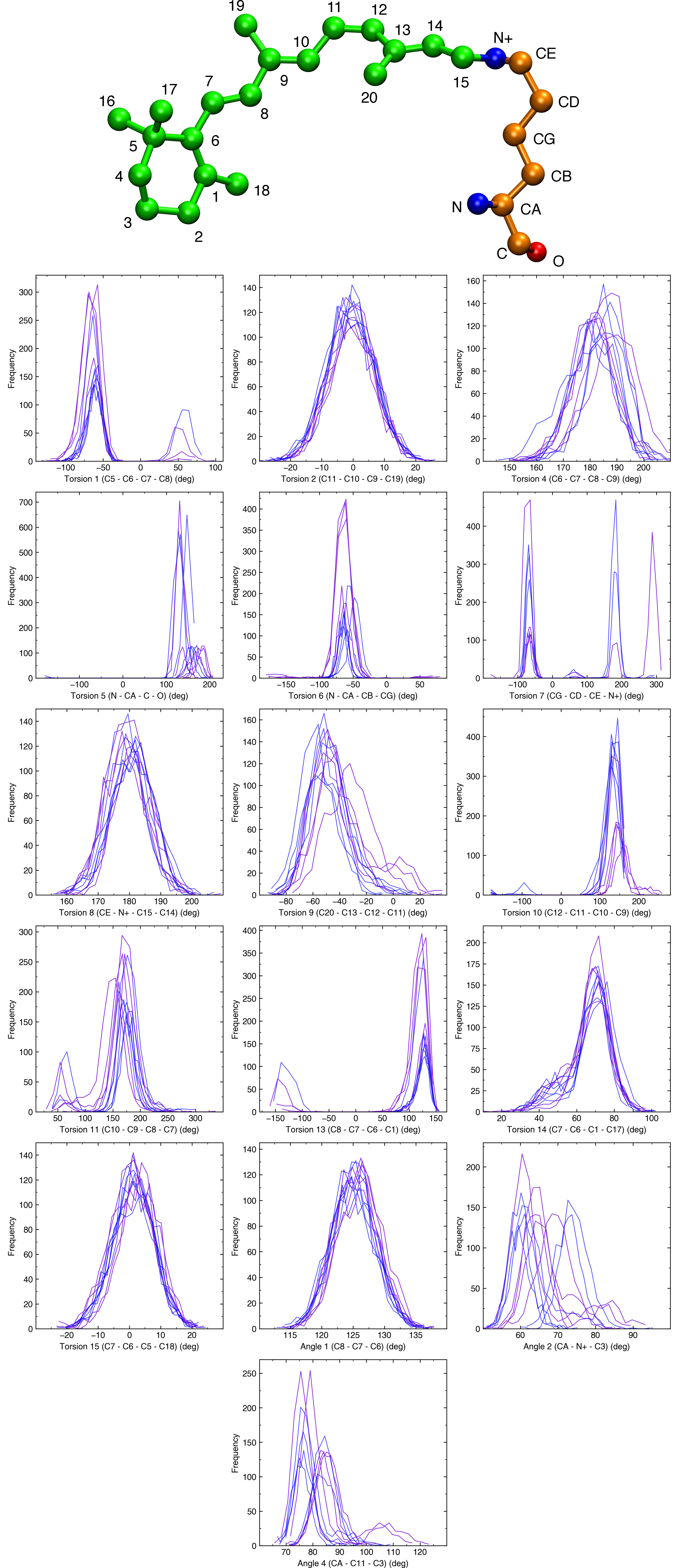

Supplement: S2 Fig — Each panel represents the frequency distribution of an individual torsions and angles associated with photopigment. Blue and violet colors indicate the relative spectral peaks of absorbance of each Sws2 photopigment, corresponding to λmax values >430 nm vs. λmax values <430 nm, respectively. (TIF) [file pcbi.1008212.s002.tif]

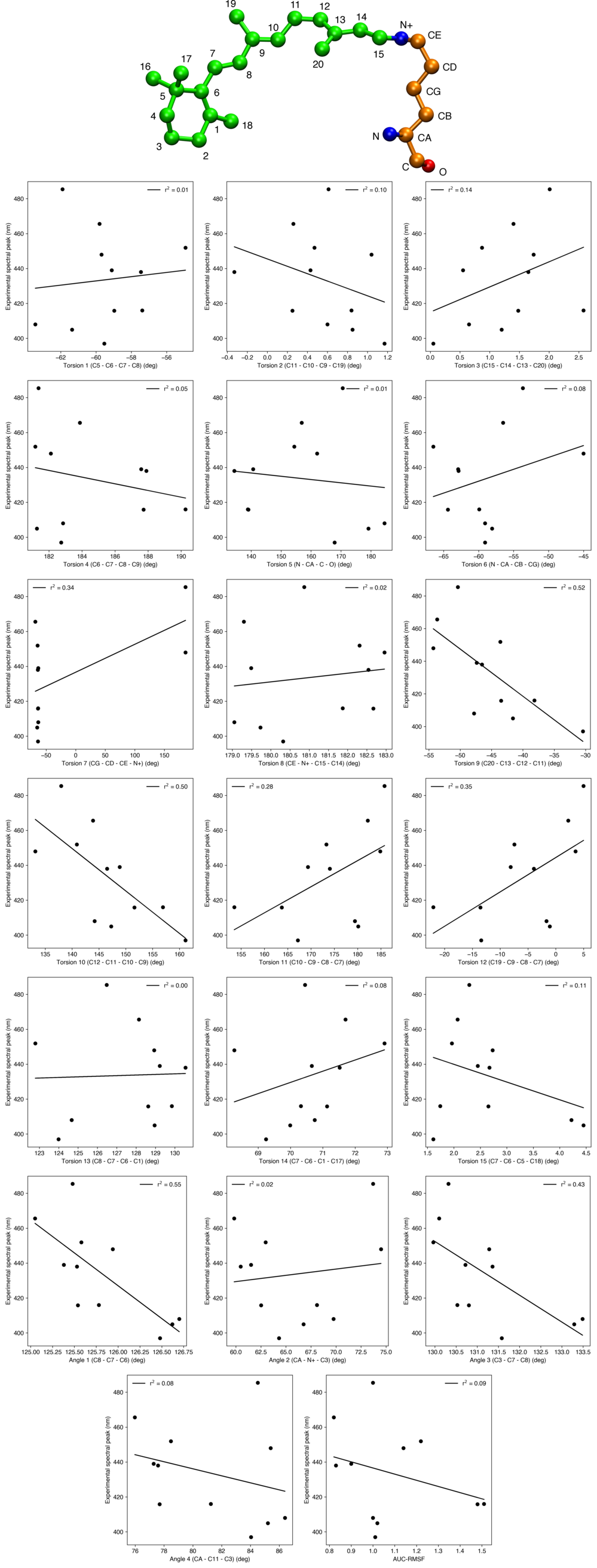

Supplement: S3 Fig — Each panel shows the relationship between the given parameter and the experimental spectral peaks of absorbance. Correlation in terms of r2 is given and the corresponding best-fit line is shown in black. (TIF) [file pcbi.1008212.s003.tif]

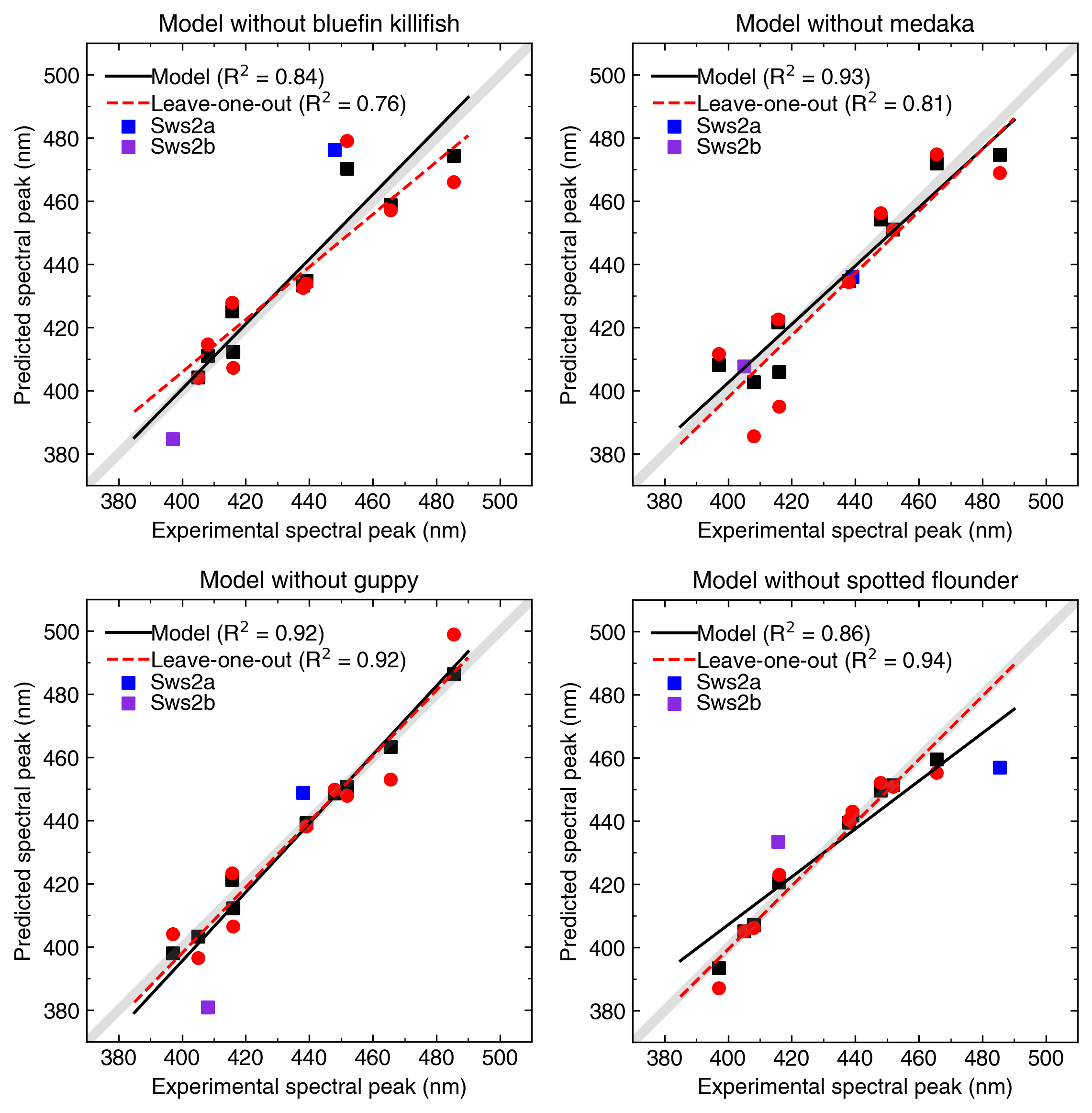

Supplement: S4 Fig — The prediction for the removed Sws2a and Sws2b photopigments are indicated by blue and violet squares, respectively. Black squares correspond to the Sws2 photopigments used in the model selection procedure with the black line indicating the R2 for the model. Red circles and dashed line correspond to the leave-one-out analysis for single photopigments included in the model. The grey line corresponds to a 1:1 relationship. (TIF) [file pcbi.1008212.s004.tif]

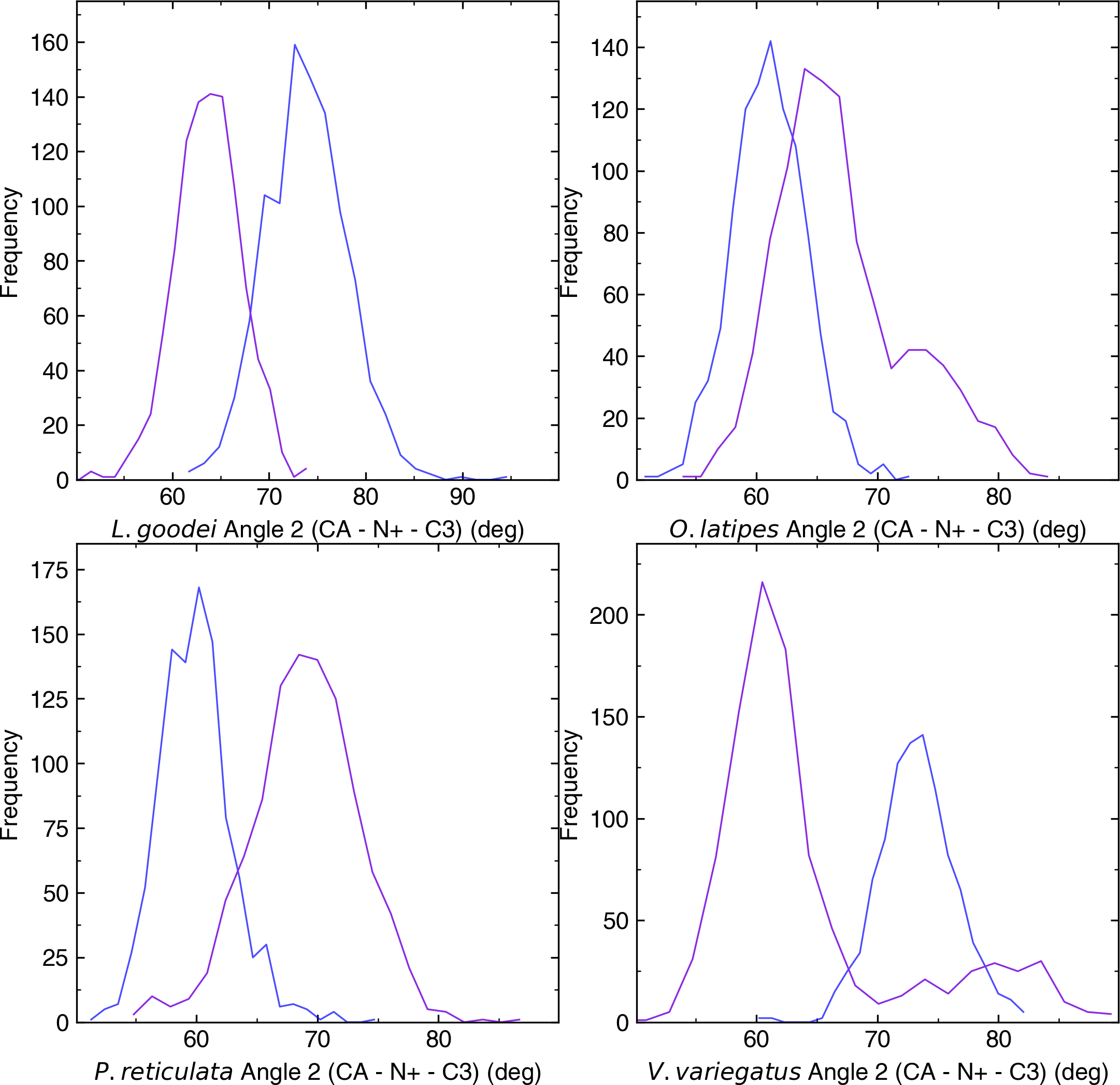

Supplement: S5 Fig — Each panel represents the frequency distribution of Angle 2 observed in individual species with Sws2a and Sws2b (L. goodei, O. latipes, P. reticulata, V. variegatus). Sws2a and Sws2b photopigments are indicated by blue and violet colors, respectively. (TIF) [file pcbi.1008212.s005.tif]
